# Tai Chi training’s effect on lower extremity muscle co-contraction during single- and dual-task gait: Cross-sectional and randomized trial studies

**DOI:** 10.1371/journal.pone.0242963

**Published:** 2021-01-22

**Authors:** Peter M. Wayne, Brian J. Gow, Fengzhen Hou, Yan Ma, Jeffrey M. Hausdorff, Justine Lo, Pamela M. Rist, Chung-Kang Peng, Lewis A. Lipsitz, Vera Novak, Brad Manor

**Affiliations:** 1 Osher Center for Integrative Medicine, Brigham and Women’s Hospital, Harvard Medical School, Boston, Massachusetts, United States of America; 2 Division of Preventive Medicine, Brigham and Women’s Hospital, Harvard Medical School, Boston, Massachusetts, United States of America; 3 Division of Interdisciplinary Medicine and Biotechnology, Beth Israel Deaconess Medical Center, Harvard Medical School, Boston, Massachusetts, United States of America; 4 Key Laboratory of Biomedical Functional Materials, School of Science, China Pharmaceutical University, Nanjing, China; 5 Center for the Study of Movement, Cognition, and Mobility, Neurological Institute, Tel Aviv Sourasky Medical Center, Sagol School of Neuroscience and Sackler Faculty of Medicine, Tel Aviv University, Tel Aviv, Israel; 6 Hinda and Arthur Marcus Institute for Aging Research, Hebrew SeniorLife, Boston, Massachusetts, United States of America; 7 Division of Gerontology, Beth Israel Deaconess Medical Center, Harvard Medical School, Boston, Massachusetts, United States of America; 8 Department of Neurology, Beth Israel Deaconess Medical Center, Harvard Medical School, Boston, MA, United States of America; University of Florida, UNITED STATES

## Abstract

**Background:**

Tai Chi (TC) mind-body exercise has been shown to reduce falls and improve balance and gait, however, few studies have evaluated the role of lower extremity muscle activation patterns in the observed benefits of TC on mobility.

**Purpose:**

To perform an exploratory analysis of the association between TC training and levels of lower extremity muscle co-contraction in healthy adults during walking under single-task (ST) and cognitive dual-task (DT) conditions.

**Methods:**

Surface electromyography of the anterior tibialis and lateral gastrocnemius muscles was recorded during 90 sec trials of overground ST (walking normally) and DT (walking with verbalized serial subtractions) walking. A mean co-contraction index (CCI), across all strides, was calculated based on the percentage of total muscle activity when antagonist muscles were simultaneously activated. A hybrid study design investigated long-term effects of TC via a cross-sectional comparison of 27 TC experts and 60 age-matched TC-naïve older adults. A longitudinal comparison assessed the shorter-term effects of TC; TC-naïve participants were randomly allocated to either 6 months of TC training or to usual care.

**Results:**

Across all participants at baseline, greater CCI was correlated with slower gait speed under DT (β(95% CI) = -26.1(-48.6, -3.7)) but not ST (β(95% CI) = -15.4(-38.2, 7.4)) walking. Linear models adjusting for age, gender, BMI and other factors that differed at baseline indicated that TC experts exhibited lower CCI compared to TC naives under DT, but not ST conditions (ST: mean difference (95% CI) = -7.1(-15.2, 0.97); DT: mean difference (95% CI) = -10.1(-18.1, -2.4)). No differences were observed in CCI for TC-naive adults randomly assigned to 6 months of TC vs. usual care.

**Conclusion:**

Lower extremity muscle co-contraction may play a role in the observed benefit of longer-term TC training on gait and postural control. Longer-duration and adequately powered randomized trials are needed to evaluate the effect of TC on neuromuscular coordination and its impact on postural control.

**Trial registration:**

The randomized trial component of this study was registered at ClinicalTrials.gov (NCT01340365).

## Introduction

Healthy gait and postural control require the effective function and coordination of lower extremity and trunk muscles [1–3]. Muscle co-contraction measured using electromyography (EMG), and defined as the simultaneous activation of agonist and antagonist muscle groups, can serve as an informative clinical marker of mobility health [4]. Relatively high muscle co-contraction is associated with joint degeneration, fatigue, reduced physical performance, and increased falls [2, 5–7], as well as a higher energy cost of walking [8, 9]. Increased co-contraction in older adults is associated with reduced joint mobility and has been hypothesized as a strategy to compensate for decreased postural control and sensory processing [10]. Muscle co-contraction has also been shown to positively correlate with aging from adulthood into senescence [11–13]. For these reasons, strategies that reduce lower-extremity co-contraction may be particularly advantageous for the promotion of safe, independent mobility and physical activity in older adults [11–13].

Tai Chi is an increasingly popular multi-modal mind-body exercise used to enhance mobility and function in older adults [14]. Tai Chi has been shown to reduce falls [15–18] and improve clinical measures of balance [15, 19–22] and gait (e.g., velocity, stride length, single-leg stance time, and stride time variability) [23–25]. Recent studies suggest that the benefits of Tai Chi’s mind-body training on gait may be more pronounced during “dual-task” challenges that require attention shifting between cognitive and motor tasks [25, 26]. Surprisingly, few studies have evaluated the potential role of lower extremity muscle activation patterns in contributing to the observed benefits of Tai Chi on mobility and postural control [27, 28].

To inform the potential of Tai Chi to effect lower extremity co-contraction, this exploratory analysis of secondary outcomes reports on the effect of both long- and short-term Tai Chi training on EMG-assessed co-contraction of the anterior tibialis and lateral gastrocnemius muscles during both undisturbed single-task, and dual-task overground walking in healthy older adults. Because previous work has shown that co-contraction of this pair of muscles is sensitive to dual-task challenges during overground walking [29], may be mediated in part by executive cognitive impairment, and that Tai Chi's benefits to gait health are more pronounced during dual-task challenges, we anticipated that: 1) Age-matched Tai Chi experts with long-term training experience would exhibit lower levels of co-contraction when compared Tai Chi naive older adults, particularly under dual-task challenges; 2) Dual-task walking would increase the levels of co-contraction more in Tai Chi naive than Tai Chi expert older adults; and 3) Tai Chi naive adults randomized to 6 months of Tai Chi training (vs. a waitlist no treatment control) would show reduced levels of co-contraction, particularly during dual-task walking.

## Methods

### Study design

The data presented here are part of a larger study evaluating physiological outcomes associated with long- and short-term Tai Chi training in healthy older adults. Measures of EMG during gait were secondary outcomes. Details of the study design and population characteristics are reported elsewhere [14, 25, 30–32] and are briefly summarized below. We employed a hybrid study design that included a cross-sectional observational study followed by a two-arm randomized clinical trial. The Institutional Review Boards at Beth Israel Deaconess Medical Center and Brigham and Women’s Hospital, Boston, Massachusetts, approved this study. The randomized clinical trial was registered at ClinicalTrials.gov (NCT01340365).

The cross-sectional study was designed to assess the association between long-term Tai Chi training on single and dual-task gait performance, including co-contraction during gait, by comparing Tai Chi-naive older adults with age-matched Tai Chi experts. The Tai Chi-naïve group was comprised of sixty healthy older subjects, age 50–79 years, who lived in the Greater Boston area and reported no regular Tai Chi practice within the past 5 years. Individuals were excluded if they had: 1) a chronic medical condition including cardiovascular disease, stroke, active cancer, neurological conditions, or significant neuromuscular or musculoskeletal conditions requiring chronic use of pain medication; 2) an acute medical condition requiring hospitalization within the past 6 months; 3) a self-reported inability to walk continuously for 15 minutes unassisted; or 4) regular participation in physical exercise on average 4 or more times per week. The Tai Chi expert group consisted of 27 subjects (age 50–79) who had been practicing Tai Chi regularly for at least 5 years prior to being enrolled (mean experience = 24.2 y). There was no limitation on the style of Tai Chi being practiced. With the exception of a regular Tai Chi practice, the other exclusion criteria and screening for the Tai Chi expert group were identical to that of the naïve group. Interested and eligible individuals were asked to sign an informed consent form.

**Fig 1:**
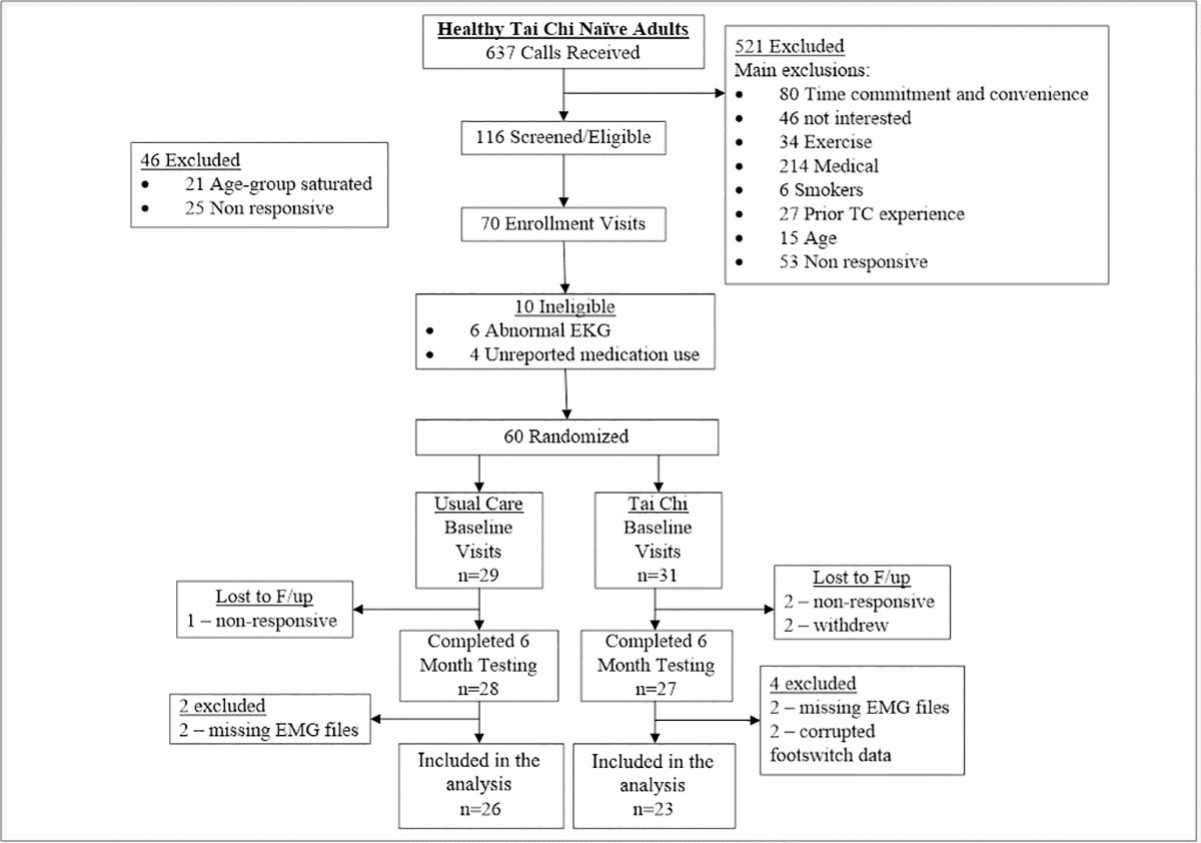
Participant flow through the randomized trial sub-study.

To assess the effects of shorter-term Tai Chi training on gait performance and lower extremity co-contraction, following baseline assessments, subjects in the Tai Chi-naïve group were randomized 1:1 to 6 months of Tai Chi in addition to their usual routine health care vs. to usual health care alone, see Fig 1. Those who were randomized to usual care alone were offered a complimentary 3 month Tai Chi course upon completion of the study. Randomization was stratified by age (50–59, 60–69, 70–79 years) and used a permuted blocks scheme with randomly varying block sizes. Randomization was performed by the study statistician. The staff members performing the gait assessments were blinded to group assignments. Tai Chi interventions were pragmatically administered at one location chosen from five pre-screened Tai Chi schools within the Greater Boston area that met certain guidelines as described elsewhere [14, 33]. Instructors were asked to teach using the same Tai Chi style, approach, and protocols that they use for non-study community participants. Study participants were asked to attend two classes per week (on average) over the 6 month intervention. They were asked to practice for at least 30 minutes on two additional days per week. Outcomes were assessed at baseline and at 6 months.

All outcomes were assessed at the Syncope and Falls in the Elderly (i.e. SAFE) laboratory at Beth Israel Deaconess Medical Center. The outcomes reported on here were part of a larger battery of tests that lasted approximately 3.5 hours per visit.

### Measurement of muscle co-contraction

Co-contraction was assessed during overground walking in a straight 5m wide hallway. Subjects were instructed to walk at their normal preferred pace and make wide turns at the end of the hallway. Trials of 90 seconds of walking were performed under two conditions; walking without a cognitive task, referred to as a single-task (ST), and walking while performing serial subtraction, referred to as a dual-task (DT). The serial subtraction task consisted of counting backward from 500 by threes. The first 5 seconds of the walks were not included in the analysis as to avoid any differences in co-contraction associated with walking initiation.

EMG electrodes were placed on the anterior tibialis and lateral gastrocnemius muscles. EMG data were sampled at 1500Hz using a Noraxon data acquisition system (Noraxon, Scottsdale, USA) with a selectable low pass filter of 500Hz. The EMG sensors had a 1^st^ order high pass filter set to 10Hz. Wireless force-sensitive resistor footswitches were placed under the subject’s toes and heels to record toe and heel strikes. The footswitches were also sampled at 1500Hz.

Data were processed using MATLAB (Mathworks, Natick, USA). Walking stride cycles were determined from footswitch data. A stride was defined as the time from a heel strike on a given leg to the subsequent heel strike on the same leg. Stride time variability was calculated from the stride time time-series as the coefficient of variation (%CV, 100 multiplied by the standard deviation of the stride times divided by the mean of each subject’s stride times). EMG data were bandpass filtered from 20 – 400Hz using a 4^th^ order Butterworth filter, full-wave rectified, and subsequently low pass filtered at 6 Hz using a 4^th^ order Butterworth, similar to Hallal et. al. [2]. Each stride was resampled to 1000 points. We used EMG to calculate co-contraction between two muscles during the entire walking bout by performing the following steps. First, the maximum EMG signal amplitude for each muscle group for all strides in a trial was noted and the mean of these maximums was calculated. Next, the EMG signals from each stride were averaged across all strides in a subject’s walk to get a mean EMG stride pattern. Next, the mean EMG stride pattern for each muscle group was then normalized as a percentage of the mean maximum amplitude to get the overall EMG stride pattern [29]. A co-contraction index (CCI) was then quantified based on this overall EMG stride pattern. The co-contraction index was defined as the percentage of mean total muscle activity when antagonist muscles (anterior tibialis and lateral gastrocnemius) were simultaneously activated, as derived from the following formula [2]:

$$Co-contraction\;index=2*\frac{common\;area\;A\&B}{area\;A+area\;B}*100$$

**Fig 2:**
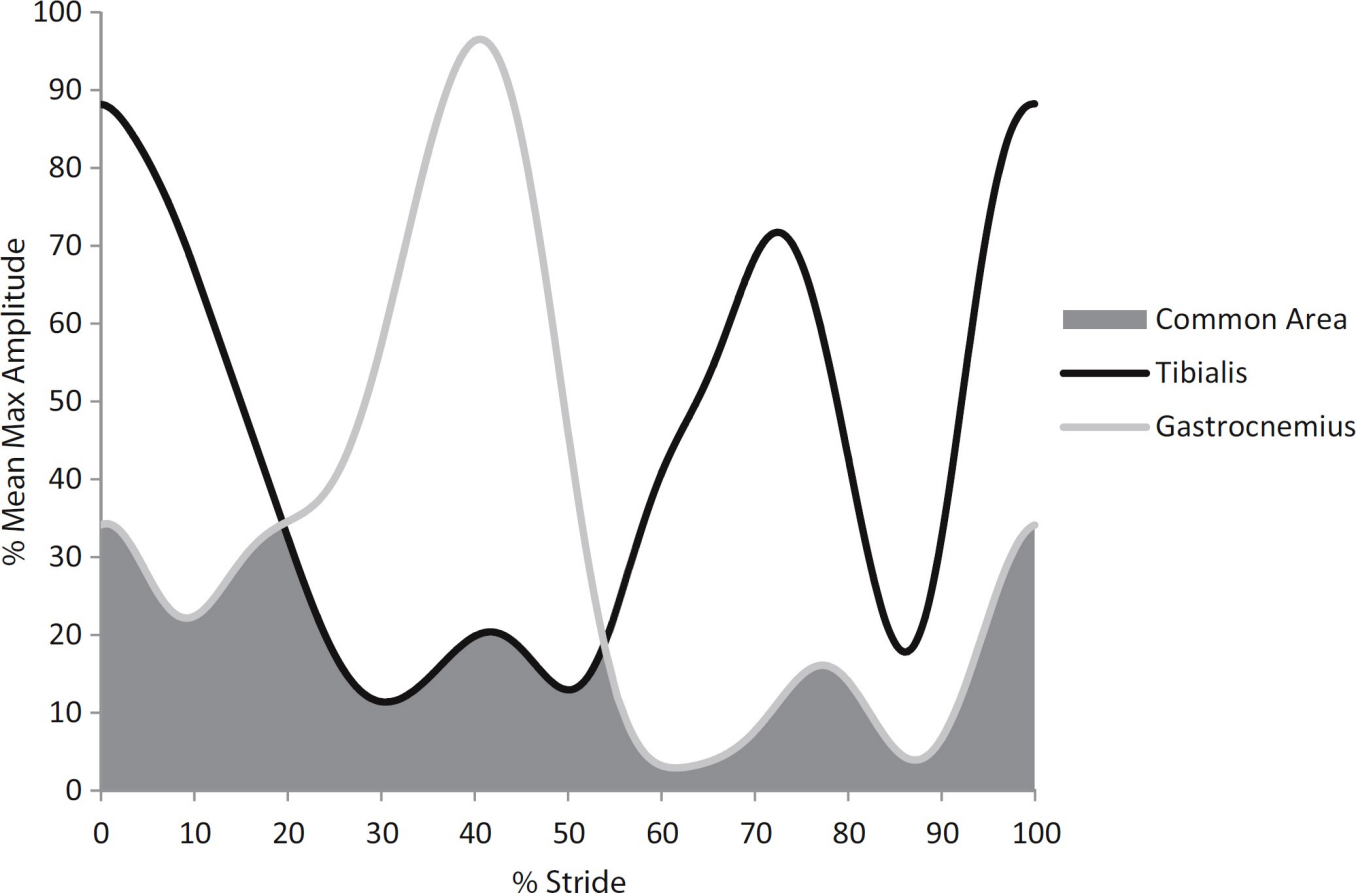
Calculation of lower limb muscle co-contraction index. The EMG signals of the anterior tibialis and lateral gastrocnemius muscles were extracted from each stride of each walking trial, then averaged, and then normalized relative to the mean of the maximum EMG amplitudes for all strides. The common area is the overlapping region under the curves of the EMG signals and represents the muscle co-contraction index. Adapted from Lo et. al. 2017 [29].

Area A was defined as the area under the EMG curve of muscle A, and area B as the area under the curve for muscle B. The common area A & B was defined as the overlapping area between muscle A and muscle B on the mean EMG curve (Fig 2).

### Statistical analysis

#### General statistical analysis considerations

Our goal in this exploratory study was not to definitively test the efficacy of Tai Chi on co-contraction, but rather to generate preliminary data relevant to our proposed mechanisms. Given that these are exploratory analyses, we anticipated that we would not have sufficient power to formally test for interactions between our exposure and task (single versus dual). Instead, we stratified our analyses by task to explore if the tasks condition may impact the exposure-outcome association.

Subjects who had missing or corrupted data at their baseline or 6 month visit were excluded from analysis, see Fig 1 for details. All analyses were performed in SAS (version 9.3, SAS Institute, Cary, NC, USA).

#### Associations between co-contraction and markers of gait performance

Associations between co-contraction and both gait speed and stride time variability across all subjects at baseline for a given task were estimated with a linear regression model. An unadjusted model along with an age, gender, BMI, education, physical activity, and hall length adjusted model are presented. Age was a priori defined as a potential confounder. Gender, BMI, education, physical activity, and an executive function Z-score were added to the model due to observed differences between the groups at baseline. Executive function was assessed with two widely used measures, Category Fluency and the Trail Making Test [34, 35]. Results of these two outcomes were combined to create a composite executive function (EF) Z-score using methods described elsewhere [31]. Midway through our study testing, we changed the hallway location in which walking was evaluated. Hallway length changed from 48m to 23m. The ratio of Tai Chi experts tested on the shorter hallways was higher than the Tai Chi naives. We also checked our assumption that these potential associations would be independent of group by comparing the slope differences between experts and naives for each task with these unadjusted and adjusted models.

#### Muscle co-contraction in Tai Chi expert vs. Tai Chi naïve adults

Cross-sectional comparisons of co-contraction between Tai Chi experts and Tai Chi naïves were assessed with linear models assuming equal variance across groups. Analyses were conducted with both unadjusted models, and with models controlling for age, gender, BMI, education, physical activity, hall length, and executive function Z-score. Reasoning for the inclusion of these confounders are mentioned in the paragraph above.

#### The effect of short-term Tai Chi training on muscle co-contraction

Longitudinal changes in co-contraction over the 6-month intervention were compared between initially Tai Chi-naïve participants randomized to Tai Chi or usual care using a random-slopes model with shared baseline. The model included fixed effect of time, time x treatment, age, and time x age and random participant-specific intercepts and slopes with unstructured covariance. The shared baseline assumption, enforced by omitting a treatment main-effect term, properly reflects the true state of the population sampled prior to randomization and has the advantage of adjusting for any chance differences at baseline [36]. Treatment-group differences and adjusted means for a participant with mean age were estimated as well as their 95% confidence intervals.

## Results

### Baseline characteristics and study flow

Tai Chi expert and naïve subjects were well matched with respect to mean age. Relative to the naïve participants, the expert group had fewer females, lower BMI, a higher number of education years, and more self-reported physical activity (Table 1). Tai Chi experts also appeared to have modestly better cognitive ability as reflected in their higher executive function Z-score. Tai Chi experts reported a mean of 24.2±12 years of Tai Chi training experience (median: 20 years, range 10–50 years). Approximately equal numbers reported Yang (n = 12) and Wu (n = 14) style Tai Chi as their primary training systems.

**Table 1:** Baseline characteristics.

		Observational groups		Randomized groups	
		Tai Chi experts (n = 26)	Tai Chi naives (n = 49)	Usual care (n = 26)	Tai Chi (n = 23)
Age		62.96 ± 7.66	64.61 ± 7.58	64.92 ± 7.55	64.26 ± 7.77
Gender	Male	12 (46.2%)	16 (32.7%)	8 (30.7%)	8 (34.8%)
	Female	14 (53.8%)	33 (67.3%)	18 (69.3%)	15 (65.2%)
BMI		22.89 ± 2.35	26.21 ± 5.34	26.51 ± 5.93	25.86 ± 4.68
Physical activity level ^a^		5.73 ± 2.03	4.35 ± 2.28	4.08 ± 2.33	4.65 ± 2.23
Education (years)		18.46 ± 3.41	16.73 ± 2.92	16.22 ± 3.06	17.32 ± 2.73
Mini mental state exam (MMSE)		29.03 ± 1.11	29.12 ± 1.07	29.15 ± 0.83	29.09 ± 1.31
Executive function Z-score ^b^		0.034 ± 0.19	-0.018 ± 0.21	-	-
Exposure to Tai-Chi hours (% of subjects)					
Compliant subjects ^c^		-	-	-	89 (65%)
Non-compliant subjects ^c^		-	-	-	45 (35%)

Unless otherwise noted values are provided as mean ± standard deviation.

^a^ 4 = Run about 1 mile/week OR walk about 1.3 miles/week OR spend about 30 min/week in comparable physical activity; 5 = Run about 1 to 5 miles per week OR walk 1.3 to 6 miles per week OR spend 30 to 60 minutes per week in comparable physical activity; 6 = run about 6–10 miles/week OR walk 7–13 miles/week OR spend 1–3h/week in comparable physical activity.

^b^ Executive function Z-score was calculated from the ratio of the standardized Trail Making and COWAT tests. More specifically the ratio of Trail Making B to Trail Making A and Category Fluency were used to generate an Executive function Z-score.

^c^ Participants that attended a minimum of 70% of all classes (two 1 hour classes per week) and completed 70% or more of prescribed home practice (two 30 minute sessions per week) were considered compliant.

Baseline characteristics for Tai Chi naïve subjects randomized to Tai Chi plus usual care or to usual care alone were comparable (Table 1). A CONSORT flowchart detailing study recruitment, randomization, and retention for the randomized trial component of the study is shown in Fig 1. Recruitment spanned from March 2011 to March 2013. All follow up procedures were completed by September 2013.

A total of 4 non-serious adverse events were reported throughout study. All events were reported by participants randomized to the Tai Chi group. Only 2 of the 4 events were determined to be related to the Tai Chi intervention (both minor musculoskeletal injuries (one wrist, one ankle)).

### Associations between co-contraction and markers of gait performance

**Fig 3:**
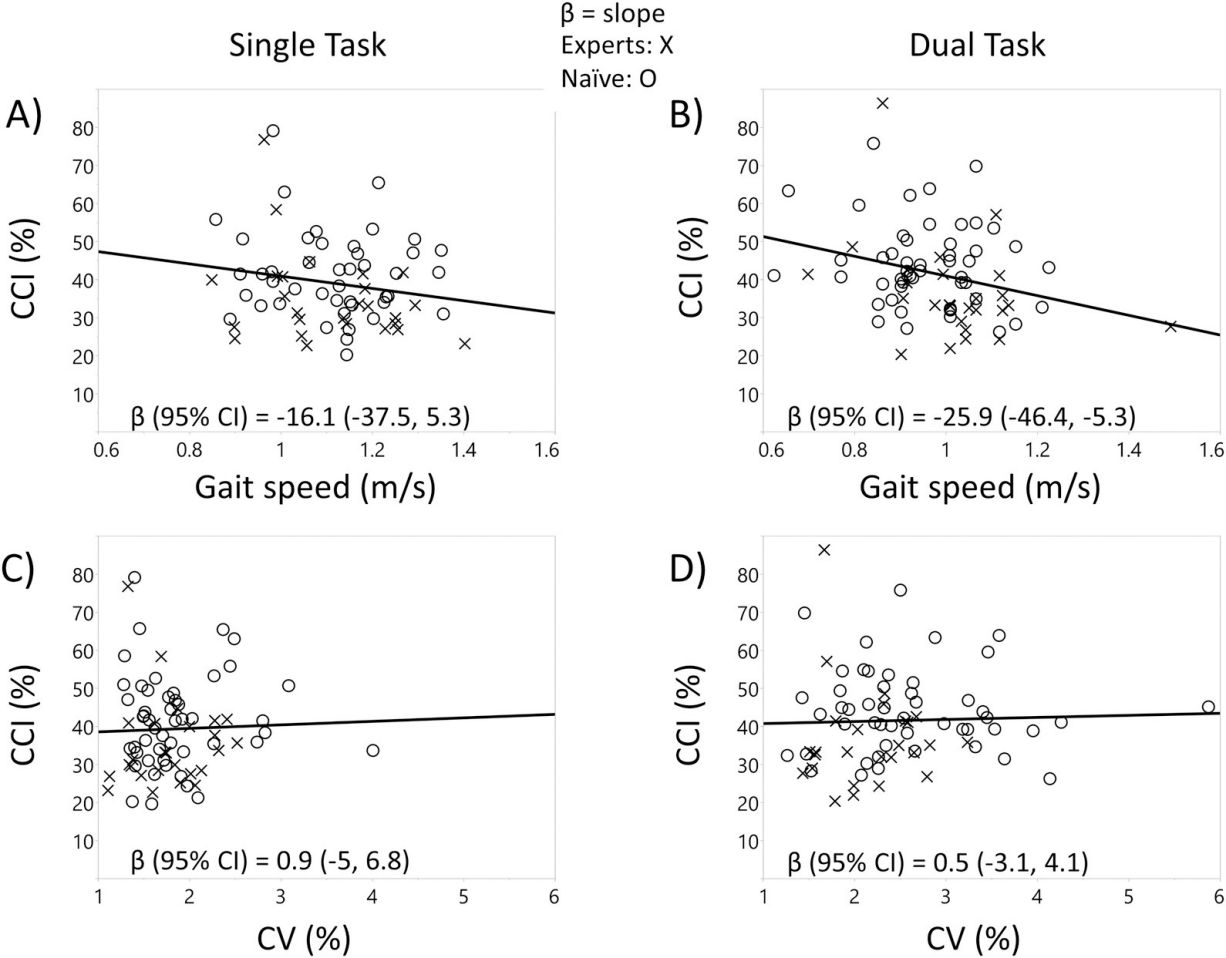
**Unadjusted associations between co-contraction vs. gait speed and stride time variability (%CV) across all subjects at baseline (solid line).** A) Gait speed vs. co-contraction index (CCI) association under the single-task condition, B) gait speed vs. CCI association under the dual-task condition, C) CV vs. CCI association under the single-task condition, D) CV vs. CCI under the dual-task condition.

Regression models revealed that participants with greater CCI exhibited slower gait speed. This negative association was apparent under the dual-task condition in both the unadjusted model (β (95% CI) = -25.9 (-46.4, -5.3)) and the age, gender, BMI, physical activity, education, hallway length, and executive function Z-score adjusted model (β (95% CI) = -26.1 (-48.6, -3.7)). β indicates the slope change in CCI per unit in gait speed (shown above) or stride time variability (further below). Gait speed was measured in meters per second. Formal definitions for CCI and stride time variability are given above in the section on the measurement of muscle co-contraction. The correlation was not as apparent, however, under the single-task condition in the unadjusted model (β (95% CI) = -16.1 (-37.5, 5.3)) or adjusted model (β (95% CI) = -15.4 (-38.2, 7.4)). Associations between co-contraction and stride time variability (%CV) were weaker under single and dual-task conditions in the unadjusted model (ST: β (95% CI) = 0.9 (-5, 6.8); DT: β (95% CI) = 0.5 (-3.1, 4.1)) and adjusted model (ST: β (95% CI) = -0.47(-7.3, 6.3); DT: β (95% CI) = 0.25 (-3.5, 4.0)). The association between CCI and either gait speed or stride time variability were similar for the Tai Chi experts and the Tai Chi naïve subjects in an unadjusted model under DT (gait speed: expert β (95% CI) = -30.8 (-66.9, 5.4), naïve β (95% CI) = -14.1 (-40, 11.8); %CV: expert β (95% CI) = -1.7 (-13, 9.7), naïve β (95% CI) = -0.6 (-4.3, 3.1)) and ST conditions (gait speed: expert β (95% CI) = -26 (-59.6, 7.6), naïve β (95% CI) = -10.2 (-37.6, 17.3); %CV: expert β (95% CI) = 0.6 (-11.7, 12.9), naïve β (95% CI) = 0.3 (-6.4, 7.1)). Similar results were seen for an age, gender, BMI, physical activity, education, hallway length, and executive function Z-score adjusted model (DT gait speed: expert β (95% CI) = -30.9 (-81, 19.3), naïve β (95% CI) = -6.5 (-35.3, 22.3); DT %CV: expert β (95% CI) = -1.5 (-14.4, 11.4), naïve β (95% CI) = -1.8 (-5.4, 1.9); ST gait speed: expert β (95% CI) = -33.6 (-74, 6.8), naïve β (95% CI) = -12.9 (-45.8, 20.1); ST %CV: expert β (95% CI) = -1.4 (-16.7, 14), naïve β (95% CI) = -0.8(-8.9, 7.2)) (see Fig 3).

### Muscle co-contraction in Tai Chi expert vs. Tai Chi naïve adults

**Fig 4:**
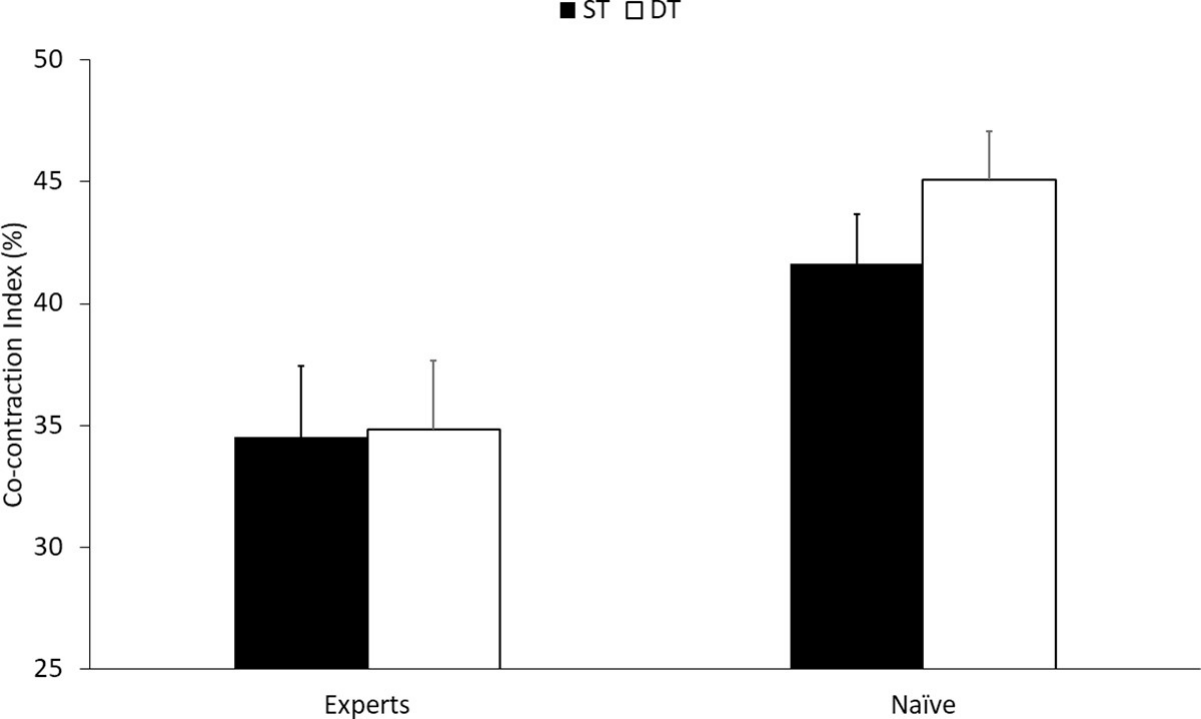
Co-contraction for age-matched Tai Chi expert and naïve older adults. Comparison between naïve and experts with age, gender, BMI, activity, education, hallway length, and executive function Z-score adjusted mean values and standard errors, shown under single-task (ST) and dual-task (DT).

Tai Chi experts (n = 26) exhibited lower CCI compared to Tai Chi naives (n = 49) under both ST and DT conditions (ST: mean difference (95% CI) = -6.5 (-12.4, -0.6); DT: mean difference (95% CI) = -7.44 (-13.2, -1.7)) in unadjusted models. Similar trends were observed in the age, gender, BMI, physical activity, education, hallway length, and executive function Z-score adjusted model (ST: mean difference (95% CI) = -7.1 (-15.2, 0.97); DT: mean difference (95% CI) = -10.1 (-18.1, -2.4)). Compared to ST walking, co-contraction trended toward being higher during DT walking in both the Tai Chi expert (ST = 34.5 ± 2.9, DT = 34.8 ± 2.8 [mean ± SE]) and Tai Chi naïve (ST = 41.6 ± 2.0, DT = 45.1 ± 2 [mean ± SE]) groups in the adjusted model (see Fig 4).

### The effect of short-term Tai Chi training on muscle co-contraction

Subjects randomized to Tai Chi plus usual care and to usual care alone both showed a reduction in co-contraction in an age adjusted model between their baseline and six-month visits under DT (Tai Chi: Δ_bl-6mo_ mean (95% CI) = -3.8 (-7.6, -0.04), usual care: Δ_bl-6mo_ mean (95% CI) = -3.7 (-7.2, -0.14)) and to a lesser extent under ST (Tai Chi: Δ_bl-6mo_ mean (95% CI) = -2.2 (-6.3, 2), usual care: Δ_bl-6mo_ mean (95% CI) = -3.4(7.3, 0.5)). This age-adjusted random-slopes model with a shared baseline did not indicate any differences across time between groups (ST: mean difference (95% CI) = 1.2 (-3.8, 6.2); DT: mean difference (95% CI) = -0.1 (-4.7, 4.5)) (Table 2).

**Table 2:** Co-contraction for the usual care and randomized to Tai Chi groups at their baseline and 6 month visits.

Outcome Measure	Tai Chi (n = 23)				Usual Care (n = 26)				Group x Time	
	Baseline		6 Month		Baseline		6 Month		ST	DT
	ST*	DT*	ST*	DT*	ST*	DT*	ST*	DT*	Mean difference (95% CI)	Mean difference (95% CI)
Co-contraction index (%)	41.3 (37.8, 44.9)	43.9 (40.7, 47.1)	39.2 (35.2, 43.2)	40.1 (36.4, 43.8)	41.3 (37.8, 44.9)	43.9 (40.7, 47.1)	38.0 (34.1, 41.8)	40.2 (36.7, 43.7)	1.2 (-3.8, 6.2)	-0.1 (-4.7, 4.5)

Age-adjusted random-slopes model with a shared baseline.

*ST and DT provided as age adjusted mean (95% CI)

## Discussion

Compared to healthy Tai Chi naive adults, age-matched Tai Chi experts exhibited lower CCI as measured with EMG during walking. Of note, this effect was more pronounced under dual-task conditions, including in models accounting for multiple potential confounders. Moreover, across both Tai Chi experts and naïve subjects, there was a negative association between levels of CCI and DT gait speed. In contrast to longer-term associations with Tai Chi training, shorter-term Tai Chi training had relatively smaller effects on CCI. Taken together, these preliminary results suggest that lower extremity co-contraction during overground walking may contribute to Tai Chi’s beneficial effect on gait health, and is a topic worthy of further exploration, particularly with a longer intervention than the 6 months used in this study.

Surprisingly few studies to date have employed EMG to evaluate the effect of Tai Chi on any aspect of gait or postural control [27, 37–40]. Observational studies have used EMG to characterize the activity of lower extremity muscles during the performance of choreographed Tai Chi sequences versus normal overground walking [41–43]. Not surprisingly, the less linear Tai Chi movements performed more slowly and with a relatively flexed knee and ankle stance resulted in higher levels of co-contraction [42]. Other studies have employed EMG to assess reaction to unpredictable postural perturbations, and reported that Tai Chi training was associated with shorter latencies in lower extremity muscles including the tibilais anterior and gastrocnemius [40, 44]. Gatts and Woolacott [28] conducted a randomized controlled trial evaluating short-term Tai Chi training (3 weeks) which employed an array of EMG sensors to evaluate lower extremity muscle responses during experimental balance recovery tasks (i.e., shifting force plates on heel strikes). Tai Chi subjects, but not controls, significantly reduced the tibialis anterior response time to these balance perturbations, as well as the occurrence of co-contraction of the tibilais anterior and gastrocnemius of the perturbed leg. That study also reported that Tai Chi subjects exhibited significant improvements in multiple clinical balance measures [28]. To our knowledge, our study is the first to inform how long- or short-term Tai Chi training may influence lower extremity co-contraction during single- and dual-task overground walking.

We observed that the lower levels of muscle co-contraction exhibited by Tai Chi experts, as compared to Tai Chi naïve counterparts, was more pronounced during dual-task walking. Increasing evidence from epidemiological studies and clinical trials support that cognition and gait health are interrelated in older adults [45–47]. In particular, executive function—which describes an array of higher-level cognitive functions such as attention, task switching, judgment of external and internal cues, and goal setting—is highly associated with multiple parameters of gait health including speed and stride time variability in older adults. Recent studies employing dual-task paradigms also suggest that the widely-reported benefits of Tai Chi for gait, balance and fall prevention may result, in part, from enhanced executive function and cognitive-motor integration [48–50]. More generally, studies identifying gait related risk factors for falls have reported greater discrimination during dual- vs. single-task observations [51, 52]. However, few studies to date have employed EMG to understand the effect of dual-task cognitive distractions on co-contraction of lower extremity antagonistic muscles during gait, or if Tai Chi could ameliorate the effect of a cognitive distraction on muscle coordination. Whether the differential benefits of Tai Chi for lower extremity muscle function and gait observed under single- versus dual-task are due to Tai Chi's enhancement of executive function [31, 53–55], motor control [56–58], or other processes warrants additional study.

In the cross-sectional component of this study, we observed a trend towards higher levels of co-contraction during DT vs. ST walking. Lo and colleagues also evaluated EMG response’s under dual- versus single-task walking and explored how interactions between cognitive and motor processes effect lower extremity neuromuscular function [29]. They did not observe a significant difference in co-contraction under DT vs. ST. However, paralleling our findings of an association between CCI and DT gait speed, they did report that CCI was associated with gait speed and stride time under dual-task but not single-task conditions. They also reported an association between executive function and co-contraction during both normal and dual-task walking. Given that prior studies have suggested that Tai Chi affects both cognitive and motor skills [22, 25, 31, 53, 59], further investigation into EMG outcomes (co-contraction, reaction time) with long-term Tai Chi interventions may be warranted, especially under dual-task conditions.

In contrast to cross-sectional comparisons between Tai Chi experts and naives, we observed only small effects of short-term Tai Chi training on levels of co-contraction. This observation parallels other clinical and phsyiological outcomes previously reported in the same study population [30–32]. The small effect observed in the cross-sectional comparison may be due to many factors including that 6 months of Tai Chi training is an insufficient dose to effect change. It is also possible that because our eligibility criteria, which targeted and resulted in a very healthy population, responses to Tai Chi were limited due to ceiling effects. Indeed, mean CCI values in our study population were markedly lower than those reported for the older and more frail population studied by Lo and colleagues [29].

This study has a number of limitations. As an exploratory study of secondary outcomes, sample sizes in both the cross-sectional and randomized studies were small and these analyses may be underpowered to detect differences between the groups. In the cross-sectional study, although we corrected for multiple potential confounders, observed between-group differences may have been confounded by other factors which we did not assess. In our cross-sectional comparison, we observed modest between-group differences in physical activity which were partially accounted for in adjusted models. Nevertheless, future studies should better control for overall physical activity levels. In our randomized trial, both the short-duration of exposure (6 months) as well as the very high levels of health of participants at baseline may have contributed to the null effect of Tai Chi on CCI. Finally, we only assessed EMG on one pair of lower extremity muscles and during short periods of relatively simple overground walking tasks. Assements of a larger array of lower extremity and trunk muscles during more diverse and challenging motor and cognitive tasks may better inform the effect of Tai Chi on mobility and postural control.

## Conclusions

Our results from the cross-sectional, and to a lesser extent, randomized group comparisons suggest that reduced co-contraction may play a role in the observed benefits of Tai Chi on gait and postural control. Given the exploratory nature of these analyses, larger and longer-duration randomized trials in both healthy and health challenged adult populations are needed to evaluate the effect of Tai Chi on co-contraction and its benefit on gait and postural control.

## Supporting information

S1 File CONSORT checklist. (DOC)

S2 File Original trial protocol. (DOC)

S3 File Final trial protocol. (DOCX)

## Abbreviations

TC: Tai Chi
EMG: Electromyography
CCI: Co-contraction index
ST: single-task
DT: dual-task
CV: coefficient of variation
BMI: Body Mass Index
SE: standard error
CI: confidence interval
